# Construction and analysis of pseudogene-related ceRNA network in breast cancer

**DOI:** 10.1038/s41598-023-49110-4

**Published:** 2023-12-10

**Authors:** Hossein Mohebifar, Amir Sabbaghian, Touraj Farazmandfar, Masoud Golalipour

**Affiliations:** Medical Cellular and Molecular Research Center, Golestan University of Medical Sciences, Shastkola Road, Falsafi Complex, Gorgān, 4934174611 Iran

**Keywords:** Breast cancer, Long non-coding RNAs

## Abstract

Breast cancer (BC) is one of the leading causes of cancer-related deaths in women. The present study explored the potential role of pseudogenes in BC via construction and analysis of a competing endogenous RNA (ceRNA) network through a three-step process. First, we screened differentially expressed genes in nine BC datasets. Then the gene-pseudogenes pairs (nine hub genes) were selected according to the functional enrichment and correlation analysis. Second, the candidate hub genes and interacting miRNAs were used to construct the ceRNA network. Further analysis of the ceRNA network revealed a crucial ceRNA module with two genes-pseudogene pairs and two miRNAs. The in-depth analysis identified the GBP1/hsa-miR-30d-5p/GBP1P1 axis as a potential tumorigenic axis in BC patients. In the third step, the GBP1/hsa-miR-30d-5p/GBP1P1 axis expression level was assessed in 40 tumor/normal BC patients and MCF-7 cell lines. The expression of GBP1 and GBP1P1 was significantly higher in the tumor compared to the normal tissue. However, the expression of hsa-miR-30d-5p was lower in tumor samples. Then, we introduced the GBP1P1 pseudogene into the MCF-7 cell line to evaluate its effect on GBP1 and hsa-miR-30d-5p expression. As expected, the GBP1 level increased while the hsa-miR-30d-5p level decreased in the GBP1P1-overexprsssing cell line. In addition, the oncogenic properties of MCF-7 (cell viability, clonogenicity, and migration) were improved after GBP1P1 overexpression. In conclusion, we report a ceRNA network that may provide new insight into the role of pseudogenes in BC development.

## Introduction

Breast cancer (BC) is the second most prevalent malignancy in women and the second-leading cause of cancer death, implying a pervasive, negative impact on the general population^1,2^. Despite significant developments in BC screening, diagnosis, and therapy, the patient's prognosis remains poor^3^. Several genes, such as BRCA1, BRCA2, and Her2, have been identified as causing BC^4–7^. In addition, long non-coding RNAs (lncRNAs) have recently been discovered to play an essential role in various cancers, including BC^8–12^. The competing endogenous RNA (ceRNA) mechanism is one of the significant routes of lncRNAs action in cancer development and progression. There are two primary sources of ceRNA molecules: circular RNAs and transcribed pseudogenes^13^.

Pseudogenes are a type of lncRNAs that evolved from their original genes^14^. Thousands of pseudogenes are encoded in the human genome^15^ and are differentially expressed in human malignancies^8,9,16^. Due to the high similarity to the parental gene, pseudogenes regulate parental gene expression via the (ceRNA) mechanism^17^. In the ceRNA mechanism, the pseudogene competes with the parental RNA to bind to shared miRNAs. Therefore, the pseudogene acts as a miRNA sponge and increases the expression of the parental gene.

The ceRNA mechanism is involved in regulating a variety of cancer-related genes^18–21^. Several lncRNAs and pseudogenes with ceRNA activity have been experimentally validated in BC patients. For example, glucocorticoids are used as supporting treatment of BC which counteracts cancer mechanoresponses through activation of LINC01569 lncRNA^22,23^. In addition, the LINC00589 ceRNA network controls patient’s response to trastuzumab treatment in HER2^+^ BC^24^. The CYP4Z2P pseudogene has anti-apoptotic and angiogenic properties in BC^25^. The CRYB2P1, PTENP1, and PTTG3P are active pseudogenes in BC that facilitates cancer progression^11,26–28^. Moreover, pseudogenes may contribute to the drug-resistance phenotype. For example, the FTH1P3 pseudogene induces paclitaxel resistance and is linked to the poor prognosis of BC patients^29^.

In this study, we tried to find a ceRNA network with potential oncogenic properties in BC. We have found several pseudogenes involved in the BC-related ceRNA network, including the GBP1/hsa-miR-30d-5p/GBP1P1 axis, which could potentially have prognostic value for BC patients.

## Materials and methods

### Study design and data collection

**Figure 1:**
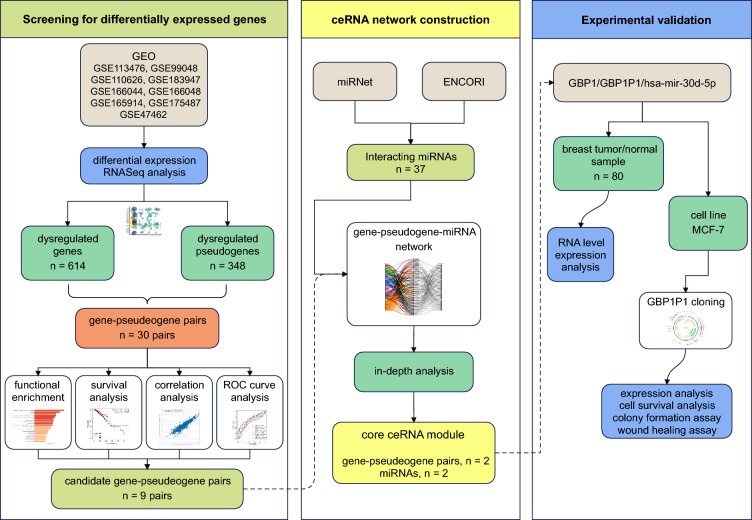
The flowchart of the study process.

This study was conducted in three steps. (1) Screening for differentially expressed genes (DGEs) in BC RNAseq datasets, (2) construction of ceRNA network using candidate genes and interacting miRNAs, and (3) experimental validation in BC patients and MCF-7 cell line. The flowchart of the study process is shown in Fig. 1.

The raw RNAseq data of BC samples, including GSE113476, GSE99048, GSE110626, GSE183947, GSE166044, GSE166048, GSE165914, GSE175487, and GSE47462 were retrieved from the GEO database. The total number of samples was 378, including 163 normal breast tissues, 209 breast cancer tissues (101 TNBC, 2 Her2+, 9 ER+, 28 early neoplasia, 24 ductal carcinomas, and 45 unclassified), as well as six breast cell lines. The breast tumor and normal adjacent samples from TCGA and breast mammary normal tissue from GTEx were used to validate the results. The interacting miRNA expression data were obtained from ENCORI (www. rnasysu.com/encori) datasets. The criteria employed for inclusion of these datasets were as follows: (i) paired normal/cancerous BC samples in each dataset, and (ii) ensuring that the samples taken before any treatments.

### RNAseq data processing and DEG analysis

The FASTQC tool (https://qubeshub.org/resources/fastqc) is employed to check raw data quality, including sequence per base quality and sequence length distribution. All reads with a Phred score lower than 30 were discarded. In addition, sequence length distribution was checked before processing data. The TRIMGALORE (https://www.bioinformatics. babraham.ac.uk/projects/trim galore) was utilized to exclude adapters and low-quality bases. The HISAT2 (http://DaehwanKimLab.github.io /hisat2) was used for aligning to the human reference genome (GRCh38). In addition, the annotation file was downloaded from the human genome browser at UCSC. Differentially expressed genes were found using the Deseq2 package (https://bioconductor.org/packages/DESeq2). We exclude all the reads except the results with an adj. *p*-value < 0.01. The median of expression values of the DEGs was then clustered using the k-means method. Pearson’s correlation coefficient for genes and pseudogenes was calculated for co-expression analysis.

### Functional enrichment

To further investigate the potential functions of DGEs, the BioPlanet (https://tripod.nih.gov/bioplanet/) version 19 was used. Gene Ontology (GO) and Kyoto Encyclopedia of Genes and Genomes (KEGG) pathway analysis were performed on 20 DGEs to explore the key biological functions and pathways of candidate genes. For each dataset we searched for the concordance degrees in the top GO terms. The GO terms included the Biological Process (BP), Cellular Component (CC) and Molecular Function (MF) categories. GO keywords and KEGG pathways were used at a significant level of *p* < 0.01 and the false discovery rate was set to 0.05. The results were visualized by http://www.bioinformatics.com.cn/srplot.

### Potential prognostic values of DEGs

We have used several analyses to find the hub genes, including survival analysis, co-expression of gene-pseudogene pairs, and ROC curve analysis. The RNAseq data with survival profiles of BC patients were extracted from the CGA database. The Kaplan–Meier (KM) survival analysis and log-rank tests were performed to evaluate the differences in the overall survival of DGEs, and the statistical significance was *p* < 0.05. Receiver operating characteristic (ROC) curves were drawn using the pROC package in R^30^. Roc curve was used to evaluate the predictive value of DGEs to distinguish BC samples from controls or chemotherapy responsive from no-responsive BC patients. The diagnostic effect was evaluated by area under the curve (AUC) values > 0.7. For co-expression analysis, the correlation of gene and pseudogene expression in BC patients was retrieved from ENCORI (https://rnasysu.com/encori).

### Target gene-miRNA prediction

Candidate gene-pseudogene pairs were used to screen related miRNAs based on experimentally-validated interactions. At first, the gene-miRNA interactions were collected from miRNet^31^. Then the StarBase was used to find associated pseudogenes^32^.

### Construction of ceRNA network and in-depth analysis

The candidate hub gene-pseudogene pairs and interacting miRNA were used to construct the ceRNA network. A matrix plot of correlations between gene-pseudogene pairs and miRNAs was used to construct a tripartite ceRNA network. The interaction of gene-miRNA-pseudogene was demonstrated by an alluvial plot.

Based on the constructed ceRNA network, an in-depth analysis was performed to find the core module of the ceRNA network. The gene-pseudogene-miRNA interaction scores were estimated according to the expression patterns and correlations. The inclusion criteria were a simultaneous negative correlation between miRNA expression and gene-pseudogene pairs in BC. Clinical information (including age, menopause status, metastasis, tumor stage, and tumor subclass) was used to compare gene-miRNA-pseudogene axes in the core ceRNA module.

## Experimental validation

### BC sample collection

For experimental validation, we collected 80 BC samples (40 breast tumors and 40 surrounding tumor-free margins) during surgery. The sample collection and experimental procedures were approved by the Golestan University of Medical Sciences Ethics Committee (IR.GOUMS.REC. 1398.009). All experiments were performed in accordance with relevant guidelines and regulations. The MCF7 cell line was used for GBP1P1 cloning and expression analysis.

### Expression of GBP1/hsa-miR-30d-5p/GBP1P1 axis in BC patients

Total RNA was extracted using TRIZOL (Gibco, Life Technologies, Carlsbad, CA, U.S.A), and cDNA was synthesized using the first strand cDNA synthesis kit (Thermo Fisher Scientific, Waltham, MA, U.S.A) from 1 µg of RNA. Relative levels of GBP1 and GBP1P1 expression were assessed using quantitative real-time PCR. The PSMB2 was used as an internal control. The sequence of primers is shown in Supplementary table S1. The PCR conditions were: 95 °C for 30 s, followed by 40 cycles of 95 °C for 5 s and 60 °C for 30 s. Relative levels of expression were calculated using the calibrator-normalized method.

### MCF-7 cell culture and GBP1P1 overexpression

The MCF7 cells were cultured in a DMEM medium with 10% FBS (Biosera, Shanghai, China). The pEGFP-C1 plasmid, harboring GBP1P1 pseudogene, was introduced into the MCF-7 for GBP1P1 overexpression. First, the vector was transformed into a competent DH5-α cell. The calcium phosphate method was used for transfection. Briefly, MCF-7 cells were plated onto 6-well plates 4–6 h before transfection. 3–5 µg of plasmid were mixed in HBS (2X) and calcium chloride 2 M solutions and filter sterilized. The mixture was sprinkled slowly on the cells and incubated at 37 °C. The transfected cells were examined under fluorescence microscopy after 24 and 48 h. Cell cultures were treated with 400 µg/ml of Neomycin G418 (Sigma-Aldrich, St. Louis, USA) for three days and harvested for further evaluation. The MCF7 cell transfected with empty pEGFP-C1 vector was used as control.

### Flow cytometry and viability assay

For cell viability assay, the pseudogene-harboring MCF-7 cells were plated onto 96-well plates and incubated overnight at 37 °C. After 24 h, 20 μl of MTS reagent was added, and plates were incubated for 4 h at 37 °C. The survival of cells was calculated according to the percentage of cell proliferation.

Flow cytometry was used to evaluate the early apoptosis in pseudogene-containing cells. Briefly, 1 × 10^6^ cells were seeded in 6-well plates for 48 h. Then, cells were washed with ice-cold PBS, trypsinized, and centrifuged at 4 °C for 4 min. Then 5 μl Annexin V-FITC (Invitrogen, USA) and 5 μl propidium iodide (PI, Invitrogen, Carlsbad, CA, USA) were added into each well. After 15 min, 400 μl cold binding buffer was added. Finally, the early apoptotic rate was measured using BD Accuri C6 Flow Cytometer (BD Biosciences, Dubai, UAE).

### Clonogenic assay

The clonogenic assay was performed on a 6-well plate. The GBP1P1-harboring MCF-7 was incubated overnight at 37 °C. Colonies were fixed with 25% methanol and stained with crystal violet for 30 min. Then, colony areas were calculated using the colony area plugin of ImageJ software^33^.

### Wound healing assay

The scratch test was performed to test the migration potential of GBP1P1-harboring cells. Briefly, cells were plated in 12-well plates, and a scratch with a pipet tip was made through the plate. Cells were washed with PBS, then RPMI 1640 medium was added in each well. The cells were photographed at 0, 6, 12, 18, and 24 h after wounding. Images were analyzed using TScratch software^34^.

### Statistical analysis

To estimate the difference between groups, various statistical tests, including paired *t*-test, Wilcoxon test, and Kruskal–Wallis test, were used for relevant analysis. For DEG profiles, the differences between groups were calculated according to *p*-values and false discovery rate (FDR). The ceRNA network is visualized by RAWGraph 2.0 (https://app.rawgraphs.io/) and Cytoscape 3.10.0^35^. The qPCR data were analyzed using the calibrator-normalized method. Data are expressed as mean ± standard error of the mean (SEM). All statistical analyses were performed using GraphPad Prism 9.0 (GraphPad Software, La Jolla, USA) and R programming language (version 4.3.0).

## Results

### The landscape of gene-pseudogene dysregulation in BC

**Figure 2:**
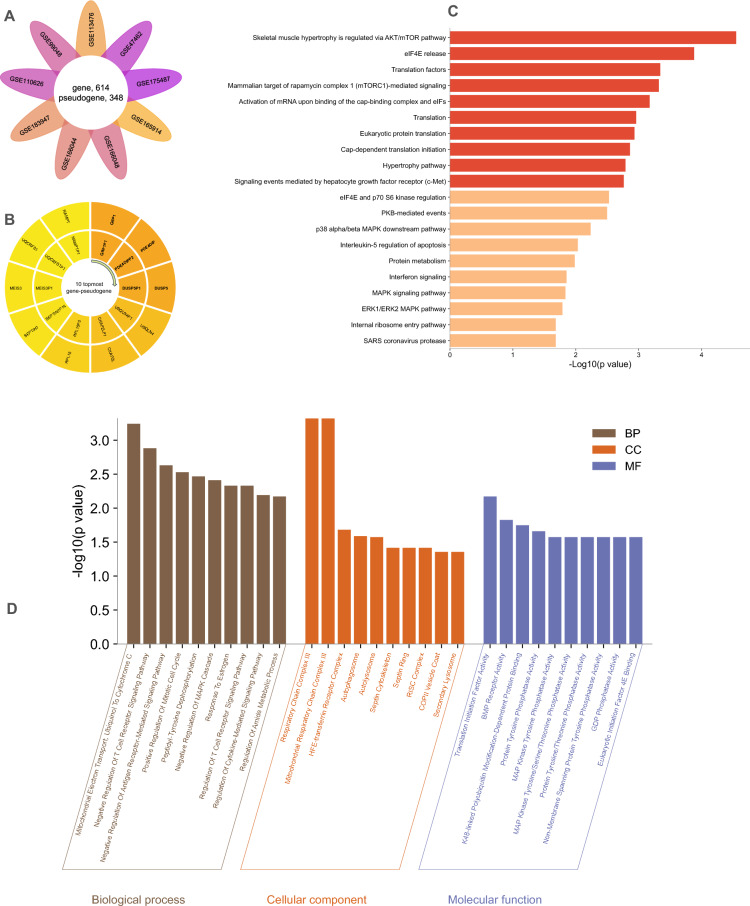
The landscape of gene-pseudogene dysregulation in breast cancer. (**A**) Flower plot diagram showing common differentially expressed genes across 9 breast cancer datasets. (**B**) gene-pseudogene pairs with the highest correlation coefficients and best *p* values. (**C**) and (**D**) Functional enrichment analysis of the gene-pseudogene pairs. Several cancer-related pathways and mechanisms were enriched in KEGG pathways and GO terms.

To identify the common dysregulated gene and pseudogenes in BC, we performed a differential gene expression analysis on nine GEO datasets (Fig. 1). As a result, we have found 962 common transcripts with significant *p* values (including 614 genes and 348 lncRNAs) in nine BC datasets (Fig. 2A). Most of the DGEs were found in more than two datasets. Then gene-pseudogene pairs were selected according to the correlation coefficient. We have found 30 gene-pseudogene pairs with significant correlation in BC datasets. GBP1-GBP1P1, PDE4DIP-PDE4DIPP2, and DUSP5-DUSO5P1 have the highest correlation coefficients and best *p* values compared to other gene-pseudogene pairs (Fig. 2B).

### Functional enrichment

Enrichment analyses were performed to investigate the biological function of differentially expressed genes (DEGs) in BC. The results of pathway analysis have shown that significant cancer-related (AKT/mTOR and c-Met pathways) and translation-related mechanisms (eIF4E release, translation factors) were enriched (Fig. 2C and D). These pathways are essential in cell proliferation, indicating their potential role in BC tumorigenesis. According to the GO terms, DEGs are primarily engaged in biological processes (mitochondrial electron transport), cellular components (respiratory chain complex III), and molecular functions (translation initiation factor activity). In molecular function, GO terms confirm the role of cancer-related pathways and translation mechanisms (Fig. 2D). Detailed results are presented in supplementary file 2.

### Screening for hub genes

**Figure 3:**
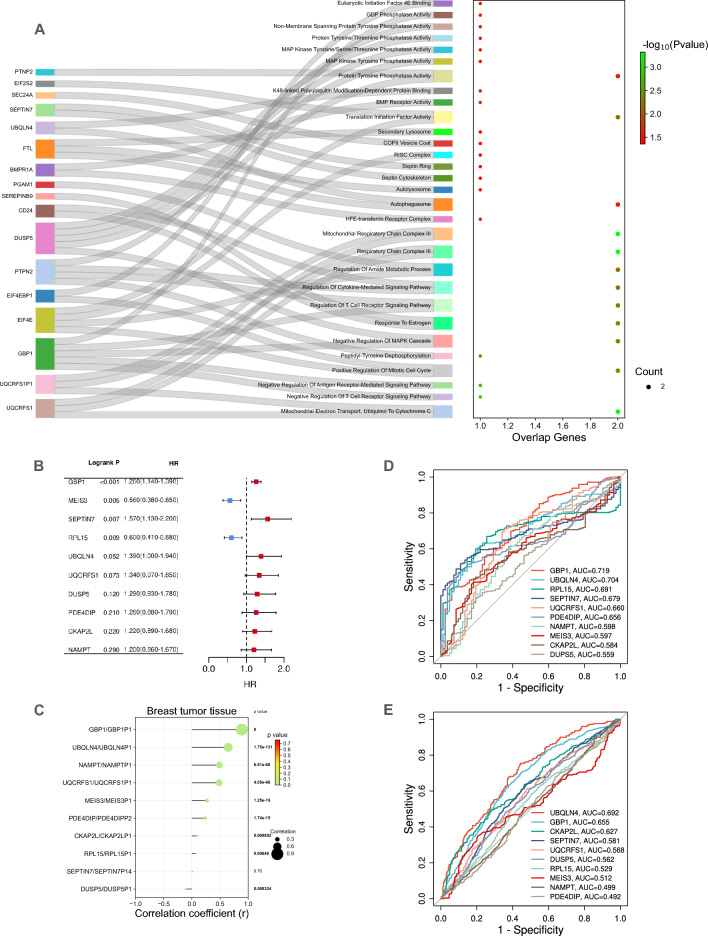
Screening for candidate Hub gene-pseudogene pairs. (**A**) The Sankey plot of genes that were involved in each enriched pathway. The dot plot shows the number of overlap genes in each pathway. The color of the dots shows the *p*-value. (**B**) Survival analysis of candidate genes in breast cancer. The Cox proportional regression analysis of hazard ratio (HZ) with 95% confidence interval. The dot shows HR, and the color shows the *p*-value. (**C**) Correlation between gene and pseudogene in breast cancer. The best correlation was observed in the GBP1-GBP1P1 pair. (**D**) and (**E**) ROC curves. The AUC was calculated for each gene to evaluate the predictive power to distinguish the breast tumor from normal (**D**) or responsive to chemotherapy from non-responders (**E**). GBP1 had the best discriminatory power in both groups.

A connection map between GO molecular function terms and genes was constructed to identify the hub genes, which depicts the number of overlapped genes with *p*-values in each pathway (Fig. 3A). According to this map, the GBP1 has the highest connections with the best *p* values. Then, the potential prognostic values of DEGs were evaluated (Fig. 2B–D). Based on the survival analysis, the GBP1 showed a significant correlation (HR = 1.26, *p*-value < 0.0001) to BC prognosis (Fig. 3B and supplementary Figure S1). When we evaluated the co-expression pattern of gene-pseudogene pairs in TCGA, some pairs showed high correlations and significant *p*-values (Fig. 3C and Supplementary table S2). GBP1 and its pseudogene, GBP1P1, demonstrated the highest correlation coefficient in BC (r = 0.888, *p* value < 0.0001). However, some gene-pseudogene pairs, for example, DUSP5-DUSP5P1, showed the opposite expression pattern (r = -0.108).

Next, these genes were queried for predictive value to discriminate breast tumors from normal samples using AUC calculation. According to ROC curves (Fig. 3D), GBP1 with AUC = 0.719 and UBQLN4 with AUC = 0.704 had the highest discriminatory power. Then, the same approach was used for chemotherapy responder/non-responder discrimination (Fig. 3E). Again, GBP1 and UBQLN4 could distinguish chemotherapy responder tumors from non-responder ones with nearly acceptable AUC (0.692 and 0.655, respectively). Detailed ROC curve data are presented in Supplementary file 3.

### Construction of ceRNA network

**Figure 4:**
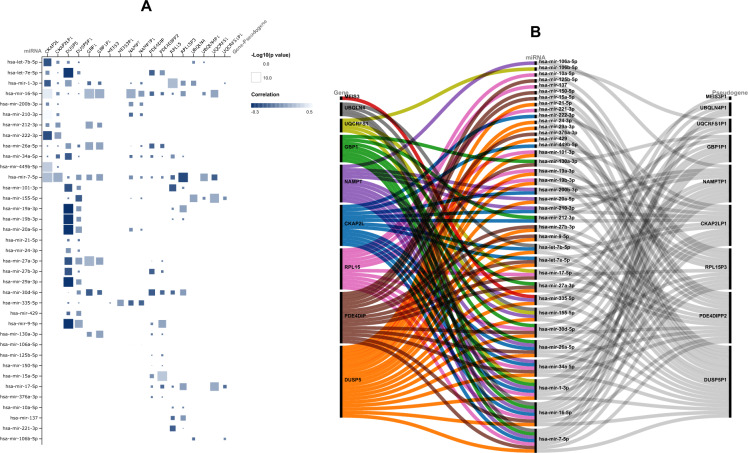
Construction of competing endogenous RNA (ceRNA) network. (**A**) Interaction plot of gene-miRNA-pseudogene. The color of the squares shows the correlation coefficient and the size of the squares shows the *p*-value. (**B**) Tripartite ceRNA network.

The hub gene-pseudogene pairs were used to screen related miRNAs according to experimentally-validated interactions retrieved from miRNet and StarBase. A matrix plot was created to demonstrate the correlation coefficients and *p* values of gene-pseudogene-miRNA interactions (Fig. 4A). Finally, with 10 hub gene-pseudogene pairs and 34 associated miRNAs, a ceRNA network was constructed (Fig. 4B). The tripartite ceRNA network shows each player in different columns.

Although only validated gene-miRNA interactions were used, this network needs to be improved. For example, some miRNAs, such as hsa-miR-7-5p and hsa-miR-16-5p, strongly correlate with several genes. However, these miRNAs positively correlate to genes unrelated to the ceRNA mechanism. Furthermore, some genes correlate negatively to several miRNAs, but their pseudogene counterpart does not follow the same pattern (DUSP5 -DUSP5P1). Therefore, we tried to find the core module of the ceRNA network according to expression patterns and correlation of miRNAs.

### The core module of the ceRNA network

**Figure 5:**
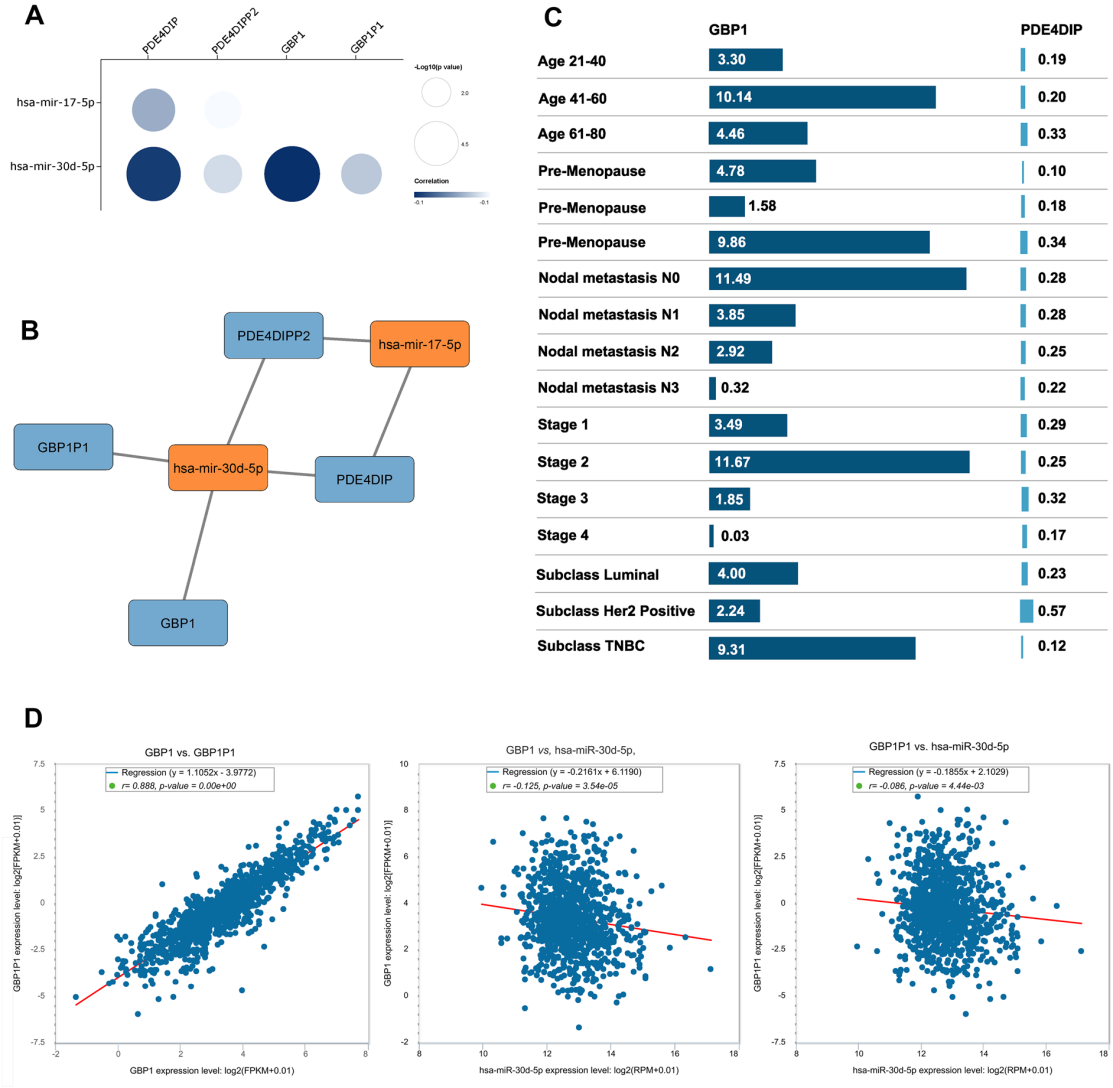
Further screening for core ceRNA module. (**A**) Matrix plot of gene-pseudogene-miRNA axes. The selection was based on the *p*-value, correlation size, and concurrent negative correlation of miRNA with gene-pseudogene pairs. (**B**) The core module of the ceRNA network with two gene-pseudogene pairs and two miRNAs. (**C**) Association between GBP1 and PDE4DIP with clinical information of breast cancer patients. (**D**) Correlation between GBP1/hsa-miR-30d-5p/GBP1P1 expressions in breast cancer.

The candidate gene-miRNA-pseudogene axes were filtered according to (1) the *p*-value and size of the correlation between miRNA and gene-pseudogene and (2) the concurrent negative correlation of miRNAs with gene-pseudogene pairs (Supplementary file 4). As a result, a core module of ceRNA was extracted (Fig. 5A and B) that contained two genes (GBP1 and PDE4DIP), two pseudogenes (GBP1P1 and PDE4DIPP2), and two miRNAs (hsa-miR-30d-5p and hsa-miR-17-5p). In this interaction network, the hsa-miR-30d-5p is negatively correlated to both GBP1-GBP1P1 and PDE4DIP- PDE4DIPP2 pairs.

We compared the correlation of BC patient clinical information (age, menopause status, metastasis, tumor stage, and tumor subclass) to GBP1 and PDE4DIP expression (Fig. 5C). GBP1 shows a more significant correlation to BC patients' characteristics than PDE4DIP. Also, we have previously (Fig. 3) shown that GBP1 has a higher prognostic value than other candidate genes. Therefore, we have finally selected GBP1/ hsa-miR-30d-5p /GBP1P1 axis for experimental validation.

### GBP1/hsa-miR-30d-5p/GBP1P1 axis expression in BC patients and cell line

**Figure 6:**
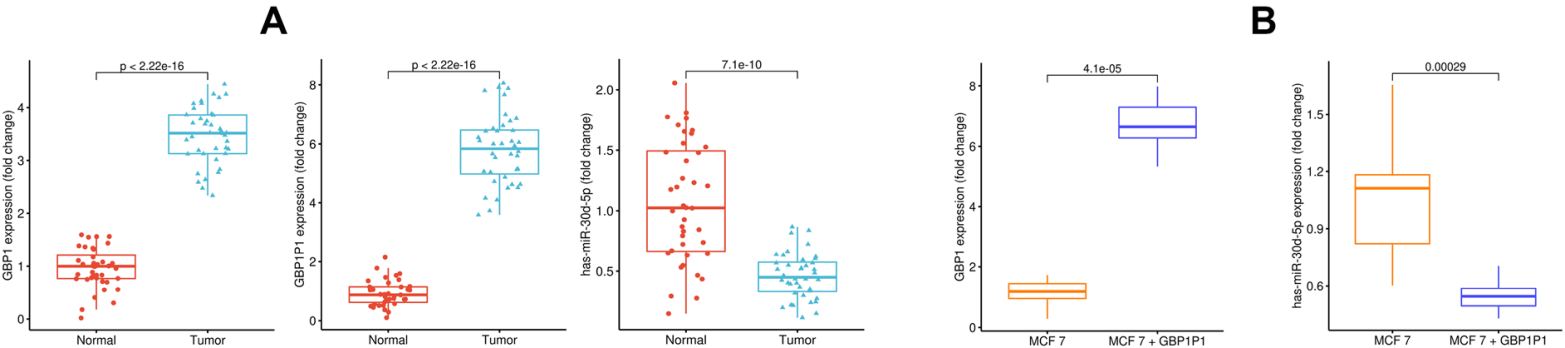
Experimental validation of GBP1/hsa-miR-30d-5p/GBP1P1 axis expression in breast cancer patient and MCF-7 cell line. (**A**) expression of GBP1/hsa-miR-30d-5p/GBP1P1 axis in breast cancer patients confirmed that GBP1 and GBP1P1 RNA level increases while the hsa-miR-30d-5p level decreases in tumor samples (*p* < 0.00001). (**B**) After GBP1P1 introduction into MCF-7, the GBP1 expression increases, and the hsa-miR-30d-5p level decreases in the GBP1P1-MCF7 cell line (*p* < 0.001).

We evaluated the expression pattern of GBP1, hsa-miR-30d-5p, and GBP1P1 in BC samples (Fig. 6A). As expected, the expression of GBP1 and its pseudogene GBP1P1 were significantly higher in tumor samples. The expression of GBP1 and GBP1P1 in tumor tissue was 3.4-fold and 5.7-fold higher, respectively. Furthermore, the expression of hsa-miR-30d-5p was lower in tumor samples compared to adjacent normal breast tissues. When the relative expression of the GBP1/hsa-miR-30d-5p/GBP1P1 axis were compared to patient’s pathological data, the expression of these genes was significantly correlated to lymph nodes metastasis, cancer stage and tumor grade (Supplementary Table S3).

When we introduced GBP1P1 into the MCF-7 cell line, GBP1 expression significantly increased in GBP1P1-harbouring cells. Also, the level of hsa-miR-30d-5p was decreased after GBP1P1 induction (Fig. 6B).

### Overexpression of GBP1P1 improves the tumorigenic properties of MCF-7

**Figure 7:**
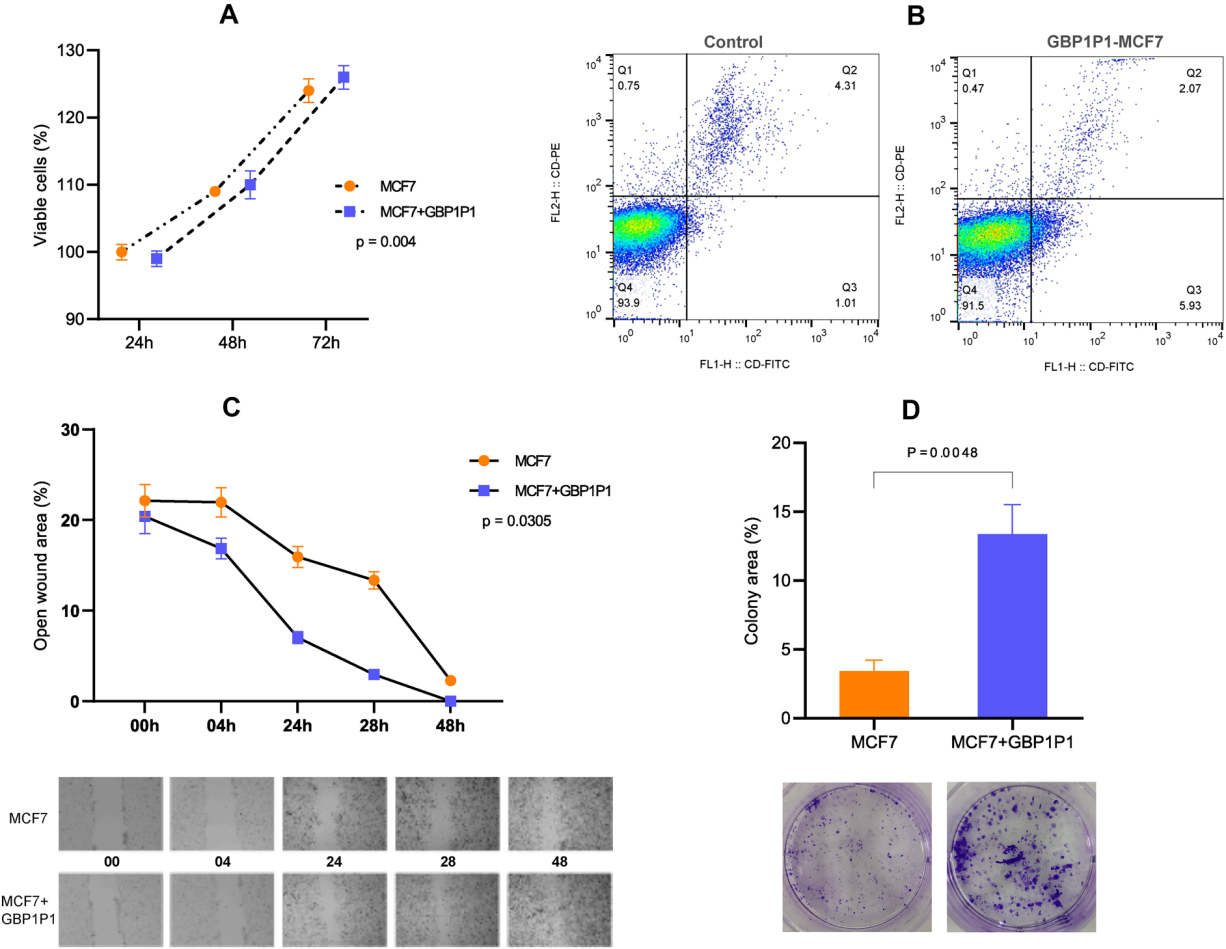
Tumorigenic properties of GBP1P1-MCF7. When GBP1P1 transfected into MCF7, the tumorigenic potential of GBP1P1-MCF7 increased. (**A**) The viability of cells was higher in GBP1P1-MCF7 cells (*p* = 0.004). (**B**) The rate of early apoptosis was higher in GBP1P1-MCF7 (5.93%), while the rate of late apoptosis was higher in control MCF-7 (4.31%). (**C**) Wound healing assay. The wound closure rate was higher in GBP1P1-MCF than control (*p* = 0.0305). (**D**) Colony formation assay. The higher colony area in GBP1P1-MCF7 cells shows more colony formation potential of GBP1P1-harboring cells.

The GBP1P1-harbouring MCF-7 cells showed more tumorigenic potential than controls. The viability of GBP1P1-MCF7 cells was higher than control MCF-7 (Fig. 7A). Also, we compared the early apoptosis of cells. Although most cells were alive in both cell lines, the GBP1P1-MCF7 cells had higher early apoptosis, while MCF-7 had higher late apoptosis rates (Fig. 7B). The early apoptosis rate in GBP1P1-MCF7 and MCF7 was 5.93% and 1.01%, respectively. In contrast, the late apoptosis rate was higher in control MCF-7 than in GBP1P1-MCF7 cells (4.31% vs. 2.07%).

In addition, GBP1P1 introduction into MCF-7 improved the wound healing properties (Fig. 7C) and colony formation potential (Fig. 7D) of GBP1P1-MCF7 cells compared to the control. In the wound healing assay, we observed faster wound closure in GBP1P1-MCF7 cells than in MCF-7 (*p* = 0.03). Moreover, in the colony formation assay, GBP1P1-MCF7 cells had more colony formation potential (*p* = 0.004).

## Discussion

Transcribed pseudogenes are genomic relicts of coding genes with the potential of serving as ceRNAs to regulate the parental gene expression level. There are several studies that explored lncRNA-related ceRNA network in BC^36^. The lncRNAs-mediated ceRNA networks regulate the expression of genes related to proliferation, drug resistance, and apoptosis and promote the development of BC. Although several studies have revealed the role of lncRNA-related ceRNA in BC, a single study (Welch et al.) was conducted on pseudogene-related ceRNA network in BC cancer^37^.

In the present study, we proposed an approach to construct a pseudogene-related ceRNA network in BC. We have found several dysregulated gene-pseudogene pairs in BC datasets. Three gene-pseudogene pairs including, GBP1-GBP1P1, PDE4DIP-PDE4DIPP2, and DUSP5-DUSO5P1 showed significant correlations. Welch et al.^37^ also reported two gene-pseudogene pairs including GBP1-GBP1P1 and SUZ12-SUZ12P1 in BC.

KEGG analysis revealed several cancer-related pathways were enriched with gene-pseudogene pairs. These pathways can be categorized into tumor-related pathways (AKT/mTOR and c-Met) and translation-related pathways (eIF4E release and cap-dependent mRNA activation factors). The enriched pathways are critical in BC development and drug resistance^38–45^. Alteration of the AKT/mTOR pathway is a usual event in BC. Approximately 70% of BC cases have distributions in AKT/mTOR pathway^39^. The c-Met pathway is a key regulator of epithelial-to-mesenchymal transition which enhances BC tumor proliferation, survival, motility, and invasion^41^. Translation initiation-related pathways are also important in BC progression. The recruitment of ribosome to mRNA is mediated by eIF4F. eIF4E is upregulated in 50% of BC and promotes tumor formation^43^.

We have extracted the core module from the ceRNA network, including two gene-pseudogene pairs and two miRNAs. The GBP1/ GBP1P1/hsa-miR-30d-5p axis showed significant correlation to clinical information. The expression levels of GBP1 and its pseudogene, GBP1P1, were significantly upregulated in tumor samples. In contrast, the hsa-miR-30d-5p level decreased in the tumor sample. Welch et al.^37^ have found a statistically significant reverse correlation between GBP1, GBP1P1, and hsa-miR-199a, which has been demonstrated to control autophagy in BC cells.

GBP1 expression has been associated with various cancers^46–56^. Knock-down of GBP1 reduces the growth of Triple-negative breast cancer (TNBC) cells^57^. GBP1 overexpression facilitate brain metastasis of BC^58^. Its pseudogene counterpart, GBP1P1, has been proposed to have a role in GBP1 regulation. The GBP1P1 has been reported as a functional pseudogene in the ceRNA network across 32 cancers^59^. Upregulation of GBP1P1 has been reported in cervical carcinoma, breast cancer, and nasopharyngeal carcinoma^60–62^. In addition, it has been proposed that the GBP1P1 expression profile could predict a complete response to chemotherapy in early BC^50^. The hsa-miR-30d-5p dysregulation has been reported in several cancers^63^. The hsa-miR-30d-5p downregulation has been reported in single hormone receptor-positive breast cancer^64^. It seems that overexpression of hsa-miR-30d-5p suppresses the PI3K/AKT Pathway. Therefore, the downregulation of hsa-miR-30d-5p leads to tumor development^65^. However, the underlying mechanism of GBP1/hsa-miR-30d-5p/GBP1P1 axis function in cancer needs further investigations.

In conclusion, we report a gene-pseudogene ceRNA network in BC, which may provide new insight into the role of gene-miRNA-pseudogene axes in BC development.

### Supplementary Information

Supplementary Information 1.

Supplementary Information 2.

Supplementary Information 3.

Supplementary Information 4.

## Data Availability

The datasets analyzed during the current study (including GSE113476, GSE99048, GSE110626, GSE183947, GSE166044, GSE166048, GSE165914, GSE175487, and GSE47462) are available in the GEO repository, [https://www.ncbi.nlm.nih.gov/geo/].
