# Supplementary material for: Construction and analysis of pseudogene-related ceRNA network in breast cancer
Source: Sci Rep. 2023 Dec 10;13:21874. doi: 10.1038/s41598-023-49110-4 (PMC10711010; doi:10.1038/s41598-023-49110-4)

**Supplementary 1**

Table S1. primer sequences for real-time PCR analysis.

| **Gene** | **Sequence** | | **Amplicon (bp)** |
| --- | --- | --- | --- |
| PSMB2 | F | ACGGCAGCAGCTAACTTCACA | 108 |
|  | R | TGGCCCTTCATGCTCATCA |  |
| GBP1 | F | CAAGGGAACAGCCTGGACAT | 107 |
|  | R | TCTGGATTCGCCATCAGTCG |  |
| GBP1P1 | F | CTGGAAGAGCAAGAGAGGACC | 109 |
|  | R | TGCCACAGTAAGCCTGTGAT |  |

Table S2. Gene-pseudogene co-expression.

| **Gene** | **Pseudogene** | **Correlation (r)** | **P-value** |
| --- | --- | --- | --- |
| GBP1 | GBP1P1 | 0.888 | 0.00E+00 |
| UBQLN4 | UBQLN4P1 | 0.646 | 1.75E-131 |
| NAMPT | NAMPTP1 | 0.49 | 6.91E-68 |
| UQCRFS1 | UQCRFS1P1 | 0.485 | 4.05E-66 |
| MEIS3 | MEIS3P1 | 0.268 | 1.25E-19 |
| PDE4DIP | PDE4DIPP2 | 0.236 | 1.74E-15 |
| CKAP2L | CKAP2LP1 | 0.1 | 8.82E-04 |
| RPL15 | RPL15P3 | 0.082 | 6.46E-03 |
| SEPTIN7 | SEPTIN7P14 | 0.009 | 7.60E-01 |
| DUSP5 | DUSP5P1 | -0.108 | 3.24E-04 |

Table S3. Clinical and pathological characteristics of BC patients and its correlation with GBP1/has-miR-30d5p/GBP1P1 expression

| Patient’s characteristics | | Number (percent) | P value | | |
| --- | --- | --- | --- | --- | --- |
|  |  |  | GBP1 | GBP1P1 | miR-30d |
| Average Tumor size | <5cm  >5cm | 26 (65%) | ns | ns | ns |
|  |  | 14 (35%) |  |  |  |
| Lymph nodes metastasis | yes  no | 22 (55%) | * | * | ns |
|  |  | 18 (45%) |  |  |  |
| Stage | I  II  III | 9 (22.5%) | ** | ** | * |
|  |  | 17 (42.5%) |  |  |  |
|  |  | 14 (35%) |  |  |  |
| Tumor grade | I  II  III | 15 (37.5%) | * | * | * |
|  |  | 13 (32.5%) |  |  |  |
|  |  | 12 (30%) |  |  |  |
| Estrogen receptor | +  - | 27 (67.5%)  13 (32.5%) | ns | ns | ns |
| Progesterone receptor | +  - | 22 (55%)  18 (45%) | ns | ns | ns |
| Histopathologic class | Ductal  Lobular | 33 (82.5%)  7 (17.5%) | ns | ns | ns |
| Molecular subtype | Luminal A  Luminal B  Basal-like  Her2+ | 16 (40%)  8 (20%)  9 (22.5%)  7 (17.5%) | ns | ns | ns |

Figure S1. The k-m plot of genes.


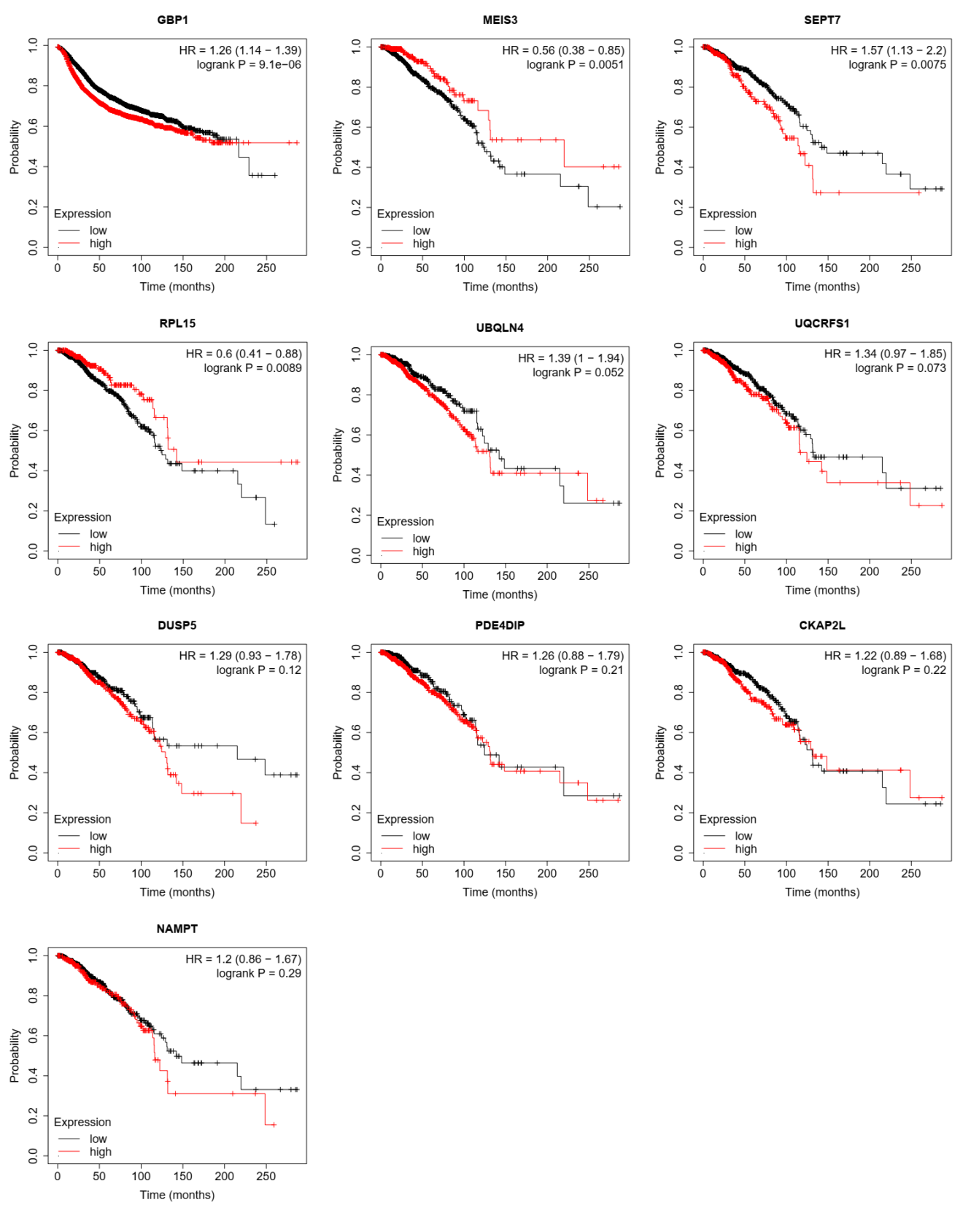

Supplement: Supplementary file 1 — Supplementary Information 1. [file 41598_2023_49110_MOESM1_ESM.docx]
